# Authors' Responses to Peer Review of “Telerehabilitation for People With Physical Disabilities and Movement Impairment: A Survey of United Kingdom Practitioners”

**DOI:** 10.2196/35845

**Published:** 2022-01-03

**Authors:** Sarah A Buckingham, Krithika Anil, Sara Demain, Hilary Gunn, Ray B Jones, Bridie Kent, Angela Logan, Jonathan Marsden, E Diane Playford, Jennifer Freeman

**Affiliations:** 1 School of Health Professions University of Plymouth Plymouth United Kingdom; 2 School of Health Sciences University of Southampton Southampton United Kingdom; 3 Centre for Health Technology University of Plymouth Plymouth United Kingdom; 4 School of Nursing and Midwifery University of Plymouth Plymouth United Kingdom; 5 Stroke Rehabilitation Royal Devon and Exeter NHS Foundation Trust Exeter United Kingdom; 6 Warwick Medical School University of Warwick Warwick United Kingdom; 7 Central England Rehabilitation Unit Royal Leamington Spa Rehabilitation Hospital Warwick United Kingdom

**Keywords:** telerehabilitation, physical disabilities, movement impairment, remote assessments, telehealth, rehabilitation, training, health care practitioners, physiotherapy, occupational therapy

*This is the authors’ response to peer-review reports for “Telerehabilitation for People With Physical Disabilities and Movement Impairment: A Survey of United Kingdom Practitioners.”*

## Round 1 Review

### Anonymous Reviewer [1]

This paper [2]: The manuscript has been well written and well organized; it needs some minor revisions.

Response: Thank you. We have responded to each of the points below.

1. It is not clear how the authors have used a combination of “opportunity” and “snowball” sampling methods considering that these are two separate methods of purposeful sampling. Additionally, it is not clear how these methods have been used, so the sampling method and justification should be explained in more detail in the Methods section; although, it has been reported as a limitation of the study.

Response: Opportunity and snowball sampling are separate but complementary methods. We used our existing contacts and networks to identify potential participants, and these participants were, in turn, asked to forward the survey to other potential participants. This has now been explained in the Methods section.

2. The first sentence of the Conclusions/Abstract is not based on the findings.

Response: One of the key findings was the increased frequency of the use of telerehabilitation since the COVID-19 pandemic (as shown in Figure 1 and supported by the qualitative findings). This finding has now been added to the Results section of the Abstract to be consistent with the rest of the paper.

3. Delete this sentence from the Methods: “No statistical correction (such as weighting of items or use of propensity scores) was used; this was not felt to be appropriate as this was not a probabilistic sample.”

Response: Sentence deleted.

4. Authors have reported their results for “pre covid-19 lock down,” “during,” and “post-covid national lock down”; this should be explained in the Methods section.

Response: As stated in the Methods section, this was a cross-sectional survey. We have added a clarifying sentence to state that the finding was based on retrospective recall in the Use of Telerehabilitation section in the Results. We have also added further detail on the lockdown restrictions imposed between March and June 2020 in the same section.

5. Providing a “Table” for reporting the results that have been reported in Figure 3 is more appropriate and readable.

Response: We feel that the figure is more engaging than a table and improves readability. In response to this comment and a suggestion made by another reviewer, we have instead added the percentages to the text in the Self-perceived Confidence and Competence section.

6. Making some policy recommendations especially for reported obstacles of using telerehabilitation strengthens the Discussion.

Response: Thank you. We agree that this strengthens the Discussion and have added a short section on Clinical and Policy Implications as recommended by other reviewers.

### Anonymous Reviewer [3]

#### General Comments

This paper reports a mixed methods survey of UK practitioners’ use of telerehabilitation for people with physical disabilities and movement impairment. It investigated practitioners’ experiences of telerehabilitation (including use, perceived benefits and obstacles, and physical outcomes assessed remotely), perceived confidence and competence, knowledge and training needs, and best practice and recommendations. It provides practical clinical recommendations for practitioners delivering telerehabilitation and has identified a number of training needs. This is very important research due to the huge uptake of virtual consultations/remote rehabilitation due to the COVID-19 pandemic and much uncertainty over its effectiveness and best practice. This paper is well written and clear to understand. I have a couple of minor comments.

Response: Thank you; we are pleased that you consider the paper to be of relevance and practical use. We have responded to each of the points below.

#### Specific Comments

##### Minor Comments

1. The Data Analysis section could include more detail regarding the qualitative analysis method used. The authors state they followed the guidance of Braun and Clarke but more detail on exactly how this was conducted would be beneficial to the readers. Relatedly, it is unclear which results they used this method for; it appears it is the *concerns of practitioners regarding the reliability and validity of remote physical assessments* (Table 4) and *practitioners’ perceived benefits and obstacles of video-based consultations* (Figure 2) sections, but this is unclear. Perhaps the authors could clarify exactly how they conducted their qualitative analysis and which data/results they used this method for.

Response: Further detail on how the qualitative analysis was carried out has been added to the Data Analysis section. Qualitative analysis was used for the following questions: reasons for not using video-based consultations, concerns regarding validity and reliability of remote physical assessments, ways of overcoming challenges; recommendations for carrying out telerehabilitation with people with physical disabilities and movement impairment, recommendations for video-based consultations with people recovering from COVID-19, open responses on information and training needs, and further comments on telerehabilitation. This is now stated in the paper.

2. There are a number of clinical practice implications from the results of this study, particularly with the recommendations for carrying out telerehabilitation in Textbox 1. It would be useful to have a clinical implications section in the Discussion, outlining how the results of this study might be useful for clinical practice.

Response: Thank you. We have added a short section on Clinical and Policy Implications to the Discussion.

### Reviewer EH [4]

#### General Comments

This paper adds to the literature base on a very timely and important topic. I appreciated how the qualitative and quantitative results are presented together to highlight each of the major findings. I have provided some comments to help improve the readability and overall quality of the paper, but in general, great work!

Response: Thank you. We are pleased that you find the paper timely and important. We have responded to each of the points below.

#### Specific Comments

##### Major Comments

1. In the Discussion regarding survey design and development, there is a discussion about how respondents could only submit responses after every relevant section was filled out. Did each question include an option of prefer not to disclose or open ended response option? If not, consider adding this in the future.

Response: We included a *prefer not to say* option for demographic questions such as gender and age but did not include this for any other questions, as we did not feel they were sensitive, and we wanted to maximize the completeness of the answers. Only the closed response questions were compulsory (and all included *other* or *none of the above* options). This has been clarified in the Design and Development section.

2. When you are including quotes in a manuscript, usually if the quote is less than 40 words, you embed it directly in the text. If it is more than 40 words, you do what you have done currently except with indentation on both sides of the quote.

Response: The formatting of quotes is in line with the editorial guidelines (ie, the use of blockquotes for quotes that are a sentence or longer).

##### Minor Comments

3. Introduction, first paragraph: “...many people received no face-to-face rehabilitation” should read “many people did not receive any face-to-face rehabilitation”

Response: Change made.

4. Introduction, second paragraph: “In response, practitioners adapted their practice” should read “In response, practitioners adapted their practices”

Response: Change made.

5. Introduction, second paragraph: “in the United Kingdom (UK) as worldwide” should read “in the United Kingdom (UK) as well as worldwide”

Response: Change made.

6. Introduction, third paragraph: “...published guidance, training and support in how to undertake...” should read “...published guidance, training and support on how to undertake...”

Response: Change made.

7. Methods, second paragraph on design and development: “This process involved informal discussions (e-mail and verbal) with specialists in rehabilitation and physical disabilities, including health and social care practitioners and academics, within and external to the project team” should read “This process involved informal discussions (e-mail and verbal) with specialists in rehabilitation and physical disabilities, including health and social care practitioners and academics within, and external to, the project team”

Response: Change made.

8. Add info regarding how long the survey took approximately to the Methods

Response: The questionnaire took approximately 15 minutes to complete. This has been added to the Design and Development section.

9. In your tables, I suggest aggregating any values that are less than 5, as this could be potentially identifying.

Response: Table 1 is the only table that contains some values with 5 or fewer respondents. We do not feel that these responses are identifying given that the survey was UK-wide and the information (eg, occupation or location) does not contain any detail. Merging the values would result in loss of information (eg, nurses and dieticians would be in the *other* category).

10. Consider reorganizing Figure 2 so that patient benefits and obstacles are side-by-side for ease of comparison

Response: Thank you. Figure 2 has been reorganized according to this suggestion.

11. You provide examples of the various types of obstacles encountered by practitioners but do not provide examples of organizational and governance obstacles; consider adding some examples of what these included.

Response: Examples of organizational and governance obstacles have been added to the Perceived Benefits and Obstacles section (eg, organizations recommending face-to-face consultations or prohibiting the use of certain technologies).

12. For Table 4, you list key themes and descriptions, which is great, but this table would benefit from an exemplar quote from each theme.

Response: A column with an exemplar quote for each theme has been added to Table 4.

13. Under “self-perceived confidence and competence,” you report “although most respondents reported that they felt confident in delivering video-based consultations, fewer had confidence in undertaking standardised clinician-rated physical assessments using this method” but do not include any actual numbers from your survey. Please add the numbers in the text rather than leaving it up to the reader to glean numbers from the figure. Additionally, you say that most respondents reported that they felt “confident,” but the questions you are discussing here have to do with proficiency/competence; consider rephrasing.

Response: Numbers and percentages have been added to the text in this section, and we feel this greatly improves readability. The terms have also been changed to reflect *proficiency* rather than *confidence* where appropriate.

14. Discussion, paragraph 5: “Understanding the actual versus perceived safety risks, and how risk averseness may impact on the type and quality...” should either read “Understanding the actual versus perceived safety risks, and how risk averseness may impact the type and quality...” or “Understanding the actual versus perceived safety risks, and how risk averseness may have an impact on the type and quality...”

Response: Rephrased according to suggestion.

### Reviewer EP [5]

#### General Comments

The content of this paper is of interest to the journal readership especially post COVID-19 pandemic and the rapid move to online practice in the rehabilitation field. It is reasonable to assume that online rehabilitation interventions are here to stay albeit to a different extent than during the pandemic. The manuscript as it stands reads well; however, the quality can be further improved by considering the following.

Response: Thank you. We are pleased that you find this paper of interest. We have responded to each of the points.

#### Specific Comments

1. Please be consistent with terminology, either the authors use “in person” or “face to face” but avoid using both terms to refer to the same method. Preferable to free text and fixed option, consider replacing with open and closed ended questions; it reads more professional.

Response: *In person* has been replaced with *face-to-face* for consistency throughout the paper. Similarly, *open response* and *closed response* questions are now referred to.

##### Main Comments

###### Title

2. Insert the word “interventions” next to Telerehabilitation.

Response: We believe that adding *interventions* would imply that the survey was only about telerehabilitation interventions and would not accurately describe the content of the paper. We explored much more than this in the survey (including experiences, attitudes, training, and assessments, not only interventions).

###### Abstract

3. The Results section could be further summarized. Suggest referring to *challenges* rather than *obstacles*.

Response: The Results section of the Abstract has been written more concisely; if you have any suggestions to improve this further, please let us know.

The terminology was discussed and agreed on by the research team prior to conducting the survey. *Obstacles* was the term used in the survey, so we would prefer to keep this term in the paper for consistency.

###### Introduction

4. There is a reasonable introduction that could be further supported with actual figures. For example, how common are the physical disabilities being referred to? Include an operational definition of physical disabilities. This would normally include motor impairment, so why does the paper refer to physical disabilities and movement impairment. I think this needs clarification supported by the literature.

Response: A reference to the Global Burden of Disease study has been added to the Introduction. A definition and distinction of impairment and disability have also been provided in paragraph 1. According to the International Classification of Functioning, Disability and Health [6], it is possible to have a physical (structural) impairment (eg, mild weakness, tremor, or loss of range/muscle length) that does not necessarily impact on function (disability).

5. It would also strengthen the rationale for the study if slightly more context were provided for key studies cited in this section [7-12].

Response: Further detail on the referenced studies has been given in the Introduction.

###### Methods

6. Design and development: the first sentence should read “findings from the scoping review...” The authors refer to “experts,” please indicate which experts these were.

Response: The first sentence has been reworded as per the suggestion. The *experts* were specialists in rehabilitation and physical disabilities, as stated in the same paragraph (this has now been clarified).

7. Second paragraph: this sentence does not read well or make sense on its own: “To maximise accuracy and completeness of data, formatting and compulsory items [13] were used in the questionnaire design.” Suggest rewriting or providing a little more explanation.

Response: The term *formatting* has been replaced with *validation*. Further explanation of compulsory items has been added.

8. Third paragraph: re: questionnaire: How long was the estimated time of completion? Could the same respondent complete it a second/multiple times? Were any measures in place to prevent this from happening? Make it clear that the questionnaire was anonymous but with an option for contact details if the respondent chose to include these.

Response: The time of completion was around 15 minutes; this has now been added. As stated in the Data Analysis section, the data set was checked for duplicate entries prior to analysis (there were none). The contact details section was optional; this is stated in the Design and Development section with more information given in the Recruitment and Data Collection section.

9. Recruitment and data collection: as a general comment, the selection criteria are not clearly explained. For example, who was classified as a rehabilitation practitioner and therefore could participate in the survey? Were there any measures in place to check that respondents were genuinely professional people (ie, verification of identity)?

Were there any exclusion criteria?

Clarify consent: was this if they returned the completed survey, then it was taken as automatic consent?

Response: Further detail on inclusion criteria has been added to this section: UK-based rehabilitation practitioners involved in rehabilitation were eligible to participate, regardless of their level of experience with telerehabilitation. This included professionals with direct patient contact, who were working in the NHS, social services, independent private, or charitable organization sectors.

We did not ask for verification of identity; as in all self-completed questionnaires, respondents can give false information about their demographics, qualifications, or any aspect of what is being asked. However, we have no reason to believe that respondents had any motivation to provide false information.

Regarding consent, an online consent form was used at the beginning of the survey (stated in this section).

10. Data analysis: Delete this sentence: “No statistical correction (such as weighting of items or use of propensity scores) was used; this was not felt to be appropriate as this was not a probabilistic sample.” It is redundant.

Response: Sentence deleted.

###### Results

11. The authors write “Of the 247 respondents, 207 (84%) reported having used video-based consultations.” The reviewer is wondering why did the other 40 not use video consultations. Was this not an inclusion criterion? Please explain.

Response: We wanted to capture the views of practitioners regardless of their level of experience with telerehabilitation (as specified in the inclusion criteria). The reasons for not using video consultations are summarized in paragraph 4 of the Perceived Benefits and Obstacles section.

12. Further down, the authors write “In free text responses, reduced travel and improved flexibility were deemed particularly beneficial for those with physical disabilities and fatigue.” Consider referring to open ended questions instead of free text. Additionally, clarify who benefited from reduced travel and improved flexibility—does this refer to professional, client, or both?

Response: *Free text responses* has been changed to *open responses*. Reduced travel and improved flexibility are potential benefits for both the patient and practitioner, but here we are referring to the most frequently selected benefits, which included reduced patient travel and convenience and flexibility of the appointment for patients.

13. The next sentence refers to multidisciplinary working. Please explain which aspects pertain to being multidisciplinary (eg, communication or decision-making).

Response: As this was not specified by the respondents who reported this as a benefit, we are unable to comment on which aspects they were referring to.

14. Figure 2: The title refers to perceived benefits, please clarify for whom? Is this written from a professional perspective, as only professionals completed this survey? It is important to make this distinction.

Response: Figure 2 refers to the benefits and obstacles of video-based consultations as perceived by practitioners. The title has been amended to clarify this.

15. Consider replacing “obstacles” with challenges, difficulties, or barriers encountered.

Response: As in comment 3, *obstacles* was the term used in the questionnaire and is unchanged for reasons of consistency.

16. Usability: Do you mean compatibility issues and unstable internet connections? If so, change in text.

Response: Examples of usability issues have been added (performance, responsiveness, and incompatibility of hardware and software).

17. It would be helpful to provide contextual examples of clients where one needs to rely on family for physical assessments. It could be that, for the client profile in question, the preferred method recommended is face-to-face—a point to comment on in the Discussion section.

Response: The following paragraph has been added to Perceived Benefits and Obstacles:

“It was recognised that telerehabilitation may not be the best option for every person or case. Examples given where practitioners felt remote consultations were less appropriate were consultations with very elderly people, people with severe cognitive, sensory or physical impairments, and cases where manual therapy such as adjustment of prostheses is required.”

This has also been referred to in the Discussion (paragraph 1).

18. Table 3: It would be helpful to include mapping of the answers to the relevant survey questions, so the reader can link the two and has a point of reference.

Response: The relevant survey questions are given in the footnote of Table 3.

19. With reference to sensory function (comment e below the table), I am finding it difficult to understand how one assesses sensory function using telerehabilitation methods accurately? Surely there must be validity and reliability issues with this method, please comment in the Discussion section.

Response: The authors feel that this is beyond the scope of the Discussion and would be more relevant in a paper that focuses solely on validity and reliability. Table 3 refers to patient-reported measures of sensory function (specifically the Dunn Adult/Adolescent Sensory Profile and the Reisman and Hanschu Sensory Integration Inventory).

20. Similarly, further down it refers to “clinician rated physical assessments.” Was there any concern for patient-reported outcomes? Especially patients who may have cognitive impairments or want to say what they think the professional wants to hear. Authors could comment on this point in the discussion.

Response: The majority of respondents commented on the validity and reliability of clinician-rated physical assessments. However, there were a small number of comments on patient-reported outcomes used remotely; a statement has been added to the Physical Outcomes Assessed Remotely section and paragraph 4 of the Discussion.

###### Discussion

21. There is a reasonable discussion in light of the findings. Further to the comments marked for the Discussion previously, the authors could also discuss/elaborate on the following.

Paragraph 5: Comment on the potential implications of avoidance in some cases as in when carrying out assessments via video or telephone

Response: The following statement has been added to paragraph 5 of the Discussion:

“Although most patients will be seen by alternative means (particularly as COVID restrictions are easing), there is a possibility that for some, this will lead to delays in diagnosis or treatment.”

22. Next, the authors make a very valid point about “Understanding the actual versus perceived safety risks” but do not elaborate. I think that this is worth further elaboration.

Response: This point has been elaborated on in paragraph 5.

23. Paragraph 6: The first line refers to “Technical and practical support from family members and carers...” What happened in cases where family/carer support was unavailable? How did professionals get around this challenge and any implications for the practice as a result?

Response: This was briefly covered in the Results section and has now been added to paragraph 6 of the Discussion.

24. Paragraph 7: Line 6 refers to training. Can the authors specify the kind of training required and which areas?

Response: This has now been elaborated on in paragraph 8 of the Discussion.

25. Last paragraph: The authors write “future surveys and qualitative studies should explore how experiences, attitudes and training needs evolve during and after the COVID pandemic.” What about duration/competence of clinical experience of the professional? Did this impact confidence? Is this a question that should be included in future surveys? Please comment.

Response: We agree that this is an important point to consider but did not include a survey question to specifically assess clinical experience/competence. This should be explored in future surveys and qualitative studies; this has been added to the end of the Limitations section.
